# Perceived Parental Support and Adolescents’ Positive Self-Beliefs and Levels of Distress Across Four Countries

**DOI:** 10.3389/fpsyg.2020.00353

**Published:** 2020-03-03

**Authors:** Yulia E. Chentsova Dutton, In-Jae Choi, Eunsoo Choi

**Affiliations:** ^1^Department of Psychology, Georgetown University, Washington, DC, United States; ^2^National Youth Policy Institute, Seoul, South Korea; ^3^Department of Psychology, Korea University, Seoul, South Korea

**Keywords:** adolescents, parental support, culture, positive self-beliefs, distress

## Abstract

Previous research has shown that parental support has beneficial effects on the psychological well-being of adolescents. Going beyond prior research, the present study made distinctions between information, emotional, and financial parental support and examined adolescents from United States (*N* = 1,002), China (*N* = 1,172), South Korea (*N* = 3,993), and Japan (*N* = 1,112). The frequency and impact of different types of perceived parental support on adolescents’ positive self-belief and distress levels have been investigated. Consistent with the existing literature, the results showed American adolescents perceived greater emotional and informational support than others, while Chinese, Korean, and Japanese adolescents perceived greater tangible support compared to American adolescents. Notably, Chinese adolescents reported higher levels of parental support than other East Asian adolescents. The perceived parental support influenced positive self-beliefs equally across cultural groups, but informational support impacted distress to a greater degree for American adolescents than East Asian adolescents. The implications of the present research are discussed.

## Introduction

Adolescence is a stage of life that is marked by increased independence from parents and heightened tension between parents and teens (see Steinberg and Morris, 2001). Despite this, parents play a critical role in scaffolding their adolescents during this period by providing them with opportunities for exercising autonomy and supporting them through trials and tribulations of puberty and early adulthood, ranging from first navigating first romantic relationships to preparing for challenging academic tests. This support is particularly important given that some teens experience drops in self-esteem (Robins et al., 2002; although also see Huang, 2010; Erol and Orth, 2011) and increases in emotional distress (e.g., depression, anxiety, Lewinsohn et al., 1994; Kessler et al., 2001; Masten, 2004; Costello et al., 2011) during this period.

Although much research has examined the impact of parental support on teens in North American cultural context, relatively little is known about cultural similarities and differences in the frequency and impact of parental support. In particular, relatively few studies have examined this topic in East Asian cultural contexts that are known to foster models of parenting and family relationships that are distinct from those fostered by North American cultural contexts (Lin and Fu, 1990; Rothbaum et al., 2000; Chao and Tseng, 2002; Su and Hynie, 2011). This study is meant to fill the void by focusing on cross-cultural similarities and differences in levels of perceived parental support and in their effects on positive self-beliefs and psychological distress of adolescents in the United States, South Korea, Japan, and China. By virtue of including three different East Asian cultural contexts, this study also aims to provide a more nuanced understanding of East Asian models of parental support. Prior to describing the study, we will briefly review extant research on perceived parental support and cultural similarities and differences in social support.

### Parental Social Support

Although support from peers gains in importance during adolescence, parental support continues to be beneficial (Robinson, 1995). By parental social support, we refer to parental provision of emotional and practical resources to their adolescent children (see Cohen, 1988). This is a broad construct that encompasses a number of different types of support, such as emotional support (or expressions of love, empathy, warmth, and concern), informational support (or information, guidance, or advice), and tangible support (or material or financial assistance) (see Cutrona and Russell, 1990). Choice of and effectiveness of these types of support is likely to depend on a number of factors such as gender of the adolescent (Robinson, 1995), his or her goals and needs (Horowitz et al., 2001), controllability of the problem (Cutrona and Russell, 1990), and recipient’s appraisal of communication (see Goldsmith, 2004).

Prior work has primarily focused on adolescents’ perceptions of their parents’ support. A sizeable body of research demonstrates that perceived parental support is linked to positive psychological outcomes, including positive self-beliefs and reduced levels of internalizing symptoms (Rollins and Thomas, 1979; Gecas and Schwalbe, 1986; Demaray and Malecki, 2002; Malecki and Demaray, 2003; Rueger et al., 2010; Auerbach et al., 2011; Stewart and Suldo, 2011; Boudreault-Bouchard et al., 2013). A number of studies suggest that parental support acts as a buffer from stress (Auerbach et al., 2011; Desjardins and Leadbeater, 2011). These effects may have positive downstream consequences for the teens’ development. For example, higher self-esteem in adolescence is known to predict lower levels of distress as well as higher levels of relational and occupational satisfaction in adulthood (Orth et al., 2012). In sum, this body of work suggests that despite adolescents’ emerging independence from their parents, parental support continues to be critical to the former psychological adjustment.

One limitation of this literature is that it tells us little about the importance of specific types of parental support. Although research indicates that distinctions between different types of support are meaningful (Robinson, 1995), relatively few studies of adolescents have examined frequency and impact of different types of social support. Most prior work conceptualized and measured support more broadly, collapsing across instances of emotional, informational and tangible support or focusing exclusively on particular types of support (e.g., emotional support, Desjardins and Leadbeater, 2011). It is possible that different types of parental support are so interrelated that this approach is sound. Indeed, one study examining middle-school-age teens reported that although perceived emotional, informational, and tangible forms of parental support jointly contributed to the teens’ well-being (Malecki and Demaray, 2003), none of these types of support did so on their own. It is also possible that additional studies may reveal that some types of support are more critical for developing adolescents’ well-being than others. For example, Robinson (1995) reported that approval and emotional support tended to impact teens’ self-worth more than instrumental support. More studies examining this question are sorely needed.

Another limitation of this literature is that much of extant research on the effects of parental support on adolescents’ positive self-beliefs and psychological distress was conducted in Western Europe and North America (for exceptions see Leung and Shek, 2018; Shek and Liang, 2018; Shek et al., 2019). North American cultural context in particular is known to be an outlier on several cultural dimensions, such as independence (Henrich et al., 2010). East Asian cultural contexts present a particularly interesting contrast to North America (see Chou, 2000) due to their emphasis on relational independence and maintenance of relational harmony (Markus and Kitayama, 1991). These different cultural models influence parental practices and beliefs (Chao, 1994, 1995). East Asian cultural contexts are less likely to promote expectations for adolescent autonomy and are more likely to stress conformity than Western European and North American cultural contexts (Trommsdorff, 1995; Fuligni, 1998). Indeed, lived family experiences in these cultural contexts support these set of emphases, with young adults from East Asian cultural contexts moving out of their parents’ homes significantly later than their European and North American counterparts (Yi et al., 1994). These cultural differences in parenting models may impact the ways in which parents support their adolescent children.

### Cultural Similarities and Differences in Social Support

Much of what we already know about culture and social support comes from studies conducted with college students and adults rather than adolescents. This work suggests that cultural contexts differ in their models of effective and appropriate social support (Xu and Burleson, 2004; Glazer, 2006; Chen et al., 2012). More specifically, research suggests that although exchanges of emotional, informational and tangible support are common across cultural contexts, East Asian contexts are less likely to emphasize emotional support than North American contexts (Xu and Burleson, 2004; Chen et al., 2012). This type of support is culturally congruent in North America as it aims to increase recipients’ levels of self-esteem and positive feelings while supporting their autonomy. Scaffolding other people’s self-esteem and positive feelings is less likely to be seen as an important interpersonal goal in East Asian cultural contexts than in North American cultural contexts (Heine et al., 1999; Brown, 2008). Instead, East Asian cultural contexts emphasize the types of support that provide practical resources, such as tangible and informational support. They also foster concerns that seeking support can be burdensome to support providers and, in response to these concerns, promote implicit forms of support (i.e., simply being together) (Taylor et al., 2004). For example, one study examined desired and actual support exchanged by married couples in the United States and China. American participants were more likely to exchange emotional support with their spouses than Chinese participants. The opposite pattern was observed for informational support, with Chinese participants wanting and getting higher levels of information and advice from their spouses than American participants (Xu and Burleson, 2004). Interestingly, despite being less common, emotional support may matter more in East Asian cultural contexts. Uchida et al. (2008) have reported that emotional support from close others was more predictive of higher levels of positive emotions and lower levels of somatic symptoms in East Asian cultural contexts (i.e., Japan, the Phillipines) than in the United States. This pattern was due to the fact that the United States, but not in the Asian cultural contexts, the impact of emotional support on well-being was mediated by its beneficial effect on self-esteem.

Few cross-cultural studies have examined parental support of adolescents. These studies report two patterns of results. On one hand, levels of expected and perceived parental support seem to depend on cultural context (Trommsdorff, 1995; Farruggia et al., 2004). For instance, adolescents in China report more parental warmth than adolescents in the United States and South Korea (Farruggia et al., 2004), suggesting that Chinese adolescents are different from Japanese adults in their perception of emotional support from parents. Meanwhile, emerging studies also suggest that the impact of parental support is relatively uniform across cultural contexts. A couple of studies examining parental social support in adolescents from East Asian cultural settings (i.e., Chinese, Vietnamese immigrants in Australia) suggest that the association of parental support with adolescents’ positive self-beliefs and internalizing symptoms that was previously observed among European and North American samples held for East Asian samples (Herz and Gullone, 1999; Chou, 2000; Kim and Rohner, 2002). One cross-cultural study compared the association of perceived parental warmth, a variable linked to emotional support, to self-esteem across the United States, China, and South Korea (Farruggia et al., 2004). The results suggested that although the levels of self-esteem differed by cultural context, the association between parental support and self-esteem was similar across contexts. Notably, this body of research is so limited by the number of studies and their methods that it is too early to draw confident conclusions based on this work.

### Current Study

More studies are needed to establish whether or not cultural models of parenting affect the nature and impact of perceived parental support. The current study compared perceived parental support and its impact on two indices of well-being, positive self-beliefs and distress, across samples of adolescents attending grades 10–12 from four different cultural contexts (United States, China, Japan, and South Korea). The present study considered positive self-beliefs encompassing self-esteem, self-efficacy, and optimism as adaptive psychological aspect of well-being and treated psychological distress encompassing negative emotional states of depression, anxiety, and anger, as a maladaptive psychological aspect of well-being. This study aimed to extend current research on parental support of adolescents in several important ways. First, it is one of very few studies to not only compare North American and East Asian cultural contexts, but to explore differences and similarities within the three different East Asian contexts. Extending the work of Farruggia et al. (2004), the current study assessed parental support in adolescents from the United States, South Korea, China, and Japan. The three interdependent East Asian cultural contexts are sometimes treated in the cultural psychology literature as if they were interchangeable in their cultural models. It is true that in the realm of parenting beliefs and practices, these contexts share strong emphases on family interdependence and Confucian models of hierarchical relationships between parents and children (see Chao and Tseng, 2002). Yet, emerging work suggest that despite these similarities, these contexts also foster some differences in cultural models of the self and relationships and in resulting beliefs about parenting. China may stand out among other East Asian cultural contexts. China, but not South Korea and Japan, is a politically communist country, with a history of collectivization and planned economy. One resulting difference that pertains to parenting is the unique impact of the long-standing One Child policy on the Chinese families (Feng et al., 2014). Chinese adolescents are more likely to be the only children and grandchildren than Korean and Japanese counterparts, leading to greater levels of parental investment, involvement and responsiveness, and, over several generations, reduced kinship networks (Lau, 1996; Short et al., 2001; Liu et al., 2010; also see Falbo and Poston, 1993). This pattern may help account for the fact that Chinese adolescents report experiencing higher levels of parental warmth than Korean adolescents in one prior study (Farruggia et al., 2004). In addition, another recent study suggests that China may differ from Japan and South Korea in its levels of independence due to its more extensive reliance on wheat farming, a farming practice that is thought to promote higher levels of relational autonomy (Talhelm et al., 2014). These cultural differences may be associated with higher levels of parental support in China than in other East Asian cultural contexts (South Korea, Japan).

Second, this study is one of the first to compare the frequency and impact of different types of perceived parental support (emotional support, informational support, and financial support, a common and quintessential type of tangible support). Although a number of prior studies have compared parenting styles or their key dimensions of warmth and control across cultural contexts (Farruggia et al., 2004; Lim and Lim, 2004), very few have focused on the impact of specific types of parental support. This is problematic because parenting styles typologies do not always show conceptual and behavioral equivalence across cultural contexts (Power et al., 1992; Kim and Rohner, 2002). Additionally, focusing on specific types of supportive behaviors may add to our knowledge as it would more directly link developmental literature to the literature on cultural similarities and differences in social support in adults.

The study tested the following hypotheses:

First, based on the literature on the impact of One Child policy on Chinese parenting (see Lau, 1996 for a review), we hypothesized that levels of perceived parental support will be higher among Chinese adolescents than Korean and Japanese adolescents. Second, based on prior work conducted with adults, we expected that levels of perceived emotional support would be higher in the United States sample than in the three East Asian samples. Conversely, we expected that levels of perceived informational and financial support would be lower in the United States sample than in the three East Asian samples.

The last set of hypotheses focused on the relationship between perceived parental support and positive self-beliefs and distress. Extant literature on this topic is relatively sparse and contradictory, leading to two alternative predictions. Some of the prior work suggests that perceived parental support would similarly predict positive self-beliefs and levels of distress across the four cultural contexts. Alternatively, other studies (e.g., Uchida et al., 2008) suggest that emotional support, in particular, would be more important for self-esteem in the United States than in Asian culture. Meanwhile, the impact of emotional support on well-being in general may be stronger for participants from East Asian cultural contexts, when adjusting for self-esteem. However, given that the previous finding came from adults and regarded perception of social support in general, it is an empirical question whether such cultural difference is also observed in the context of adolescents’ perception of parental support.

## Materials and Methods

### Sampling

The Korean sample was recruited from 24 high schools across 5 cities and 7 provinces in South Korea, between June and July of 2010. The sampling was based on the database from the Statistical Yearbook of Education published in 2009 by the Department of Education of South Korea. The 5 cities of South Korea include Seoul, Incheon, Daejeon, Gwangju, Daegu, Ulsan, and Busan and the 7 provinces include Gyeonggi, Chungcheong, Jeolla, Gangwon, and Gyeongsang.

The participants of the United States, Japan, and China were recruited between September and October of 2010. American data were collected from 13 schools across 12 cities (11 states), including Durham, North Carolina, DeWitt, New York, Kansas City, Kansas, Chicago, Illinois, Agoura Hills, California, Indianapolis, Indiana, Charlotte, North Carolina, Idaho Falls, Idaho, Liberty, Missouri, Eugene, Oregon, Gallup, New Mexico, and Tulsa, Oklahoma. Japanese data were collected from 10 schools across 8 regions including Kanagawa prefecture, Saitama prefecture, Aomori prefecture, Gifu prefecture, Ibaraki prefecture, Fukuoka prefecture, Miyagi prefecture, and Shizuoka prefecture. Chinese data were collected from 10 schools across 6 regions including Beijing, Shanghai, Xi’an, Hengyang, Yinchuan, and Liaoning. The sampling units (cities or prefectures) of these three countries were selected based on the size of the cities. The target schools of these sampling units were randomly selected from the list of high schools in these regions.

### Participants

Participants included 3,993 Koreans (51% males, 49% females), 1,002 Americans (45% males, 54.4% females), 1,112 Japanese (53.6% males, 46.3% females), and 1,172 Chinese (48.3% males, 51.4% females). There were relatively comparable proportions of students in different grades across countries. Among Koreans, there were 1,374 (34.9%) high school students in the 1^st^ grade, 1,301 (33.1%) in the 2^nd^ grade, and 1,258 (32.0%) in the 3^rd^ grade. The American sample consisted of 299 (29.8%) 10^th^ graders, 334 (33.3%) 11^th^ graders, and 369 (36.8%) 12^th^ graders. The Japanese sample consisted of 446 (40.1%) 1^st^ grade high school students, 370 (33.3%) 2^nd^ grade high school students, and 296 (26.6%) 3^rd^ grade high school students. The Chinese sample consisted of 423(36.1%) 10^th^ graders, 340 (29.0%) 11^th^ graders, and 409(34.9%) 12^th^ graders.

### Measures

The items for positive-self beliefs, distress, and parental support were developed by 10 experts in the area of adolescence research in South Korea. The items were first pilot tested with Korean adolescents, resulting in good reliabilities and validity (Choi et al., 2010). The original materials in Korean were translated into the respective languages by bilinguals with doctoral degrees and were back-translated into Korean. The original questionnaire and the back-translated questionnaire were compared and reviewed. Any discrepancies between the original and the translation were addressed by the investigators. The validity of the scales used in this study were reported in the final report submitted by the Korean National Youth Policy Institute (Choi et al., 2010). See Supplementary Appendix A for all items used.

#### Positive Self-Beliefs

Positive self-beliefs were measured with 11 items assessing levels of self-esteem, self-efficacy, and optimism (Choi et al., 2010). We conceptualized that three factors of self-esteem (e.g., “I am a valuable person”), self-efficacy (e.g., “I can do pretty much anything if I make an effort”), and optimism (e.g., “I believe my dreams will come true”) consisted the encompassing construct, positive self-beliefs. The descriptive statistics for sub-factors are included in Supplementary Appendix B. All items were measured using four-point Likert scales (1 = disagree, 2 = somewhat disagree, 3 = somewhat agree, 4 = agree). Cronbach’s alphas for the four samples were high: United States, α = 0.87; South Korea, α = 0.88, Japan, α = 0.87, China, α = 0.88.

#### Distress

Distress was measured with 11 items assessing levels of depressive symptoms, anxiety, and anger. Participants were asked, “Did you ever experience the following feelings during the past week?” and indicated the extent to which they were “gloomy,” “worried for no reason,” and “wanted to yell and throw something.” The descriptive statistics for sub-factors are included in Supplementary Appendix B. All items were measured using 4-point Likert scales (1 = disagree, 2 = somewhat disagree, 3 = somewhat agree, 4 = agree). Cronbach’s alphas for the four samples were high: United States, α = 0.88; South Korea, α = 0.90, Japan, α = 0.90, China, α = 0.89.

#### Parental Support

We measured three types of perceived parental support, informational, financial, and emotional support (Choi et al., 2010). Informational parental support was assessed with three questions asking the extent to which the adolescent perceived their parents to provide advice on career options, studies, and the right attitudes about education and life. Financial support was captured by three questions asking participants about the degree to which they perceived their parents giving them allowance and buying necessary items. Emotional support was assessed with three questions about the extent to which adolescents felt understood, and listened to about their problems, and were helped during difficult times by their parents. All items were measured using four-poin Likert scales (1 = disagree, 2 = somewhat disagree, 3 = somewhat agree, 4 = agree). Cronbach’s alphas for perceived informational support, financial support, and emotional support of the four samples were high, with the exception of lower scores for financial support, particularly in China: United States, α = 0.86, α = 0.64, α = 0.90; South Korea, α = 0.84, α = 0.69, α = 0.86; Japan, α = 0.82, α = 0.72, α = 0.88; China, α = 0.66, α = 0.52, α = 0.82. Hence, results for financial support should be interpreted with more caution, as lower reliability estimates may be limiting this scale’s validity.

## Results

### Measurement Invariance

In order to ensure that the analyses examine the same constructs across four cultural groups, multigroup confirmatory factor analyses were conducted using R lavaan package (Rosseel, 2012), following the standard procedures for testing measurement equivalence (Byrne et al., 1989). The model fit was examined with comparative fit index (CFI), Tucker-Lewis Index (TLI), root-mean-square error of approximation (RMSEA), standardized root-mean-square residual (SRMR), following Hu and Bentler’s (1999) suggestions. We treated the following criteria of multiple fit indices as indicating a reasonable model fit, CFI > 0.90, TLI > 0.90, RMSEA < 0.08, SRMR < 0.06 (Marsh et al., 2004). First, for each variable, we tested and confirmed that all items loaded on the same latent factor for four cultural groups (i.e., configural invariance). Next, we tested whether the loadings of the items on each latent factor were equal across cultures (metric invariance). Fit indices from multigroup confirmatory factor analyses for assessing the metric measurement invariance for all of the variables are presented in Table 1. Descriptive statistics and correlation coefficients of the variables in the present study is presented in Tables 2, 3.

**TABLE 1:** Fit indices from multigroup confirmatory factor analyses for assessing the metric measurement invariance of the variables in the study.

**Variables**	**X-squared**	**df**	**CFI**	**TLI**	**RMSEA**	**SRMR**
Positive self-belief	2197.312	188	0.936	0.926	0.078	0.053
Distress	3630.056	188	0.916	0.902	0.102	0.078
Emotional support	20.896	6	0.999	0.997	0.037	0.014
Informational support	9.879	6	1.000	0.999	0.019	0.011
Financial support	13.28	6	0.998	0.996	0.026	0.013

**TABLE 2:** Means and standard deviations of the variables in the study.

	**United States**	**China**	**South Korea**	**Japan**	***F*(3,7189)**
Positive self-belief	3.24 (0.55)_a_	3.12 (0.51)_b_	2.80 (0.48)_c_	2.38 (0.55)_d_	632.07***
Distress	2.07 (0.71)_c_	1.93 (0.63)_b_	1.67 (0.56)_a_	1.97 (0.75)_b_	161.49***
Emotional support	3.02 (0.92)_a_	2.99 (0.76)a	2.78 (0.71)_b_	2.78 (0.80)_b_	43.55***
Informational support	3.24 (085)_a_	2.96 (0.70)_b_	2.81 (0.72)_c_	2.57 (0.76)_d_	154.94***
Financial support	3.06 (0.80)_a_	3.38 (0.61)_c_	3.13 (0.61)_ab_	3.15 (0.70)_b_	52.94***

*** *p* < 0.001. Standard deviations appear in parentheses. Means with differing subscripts within rows are significantly different at the *p* < 0.05 based on Scheffé’s *post hoc* paired comparisons.

**TABLE 3:** Zero-order correlation coefficients of variables in four cultural groups.

	**1**	**2**	**3**	**4**
**(1) Positive self-beliefs**				
United States	–			
China				
South Korea				
Japan				
**(2) Distress**				
United States	−0.48***	–		
China	−0.29***			
South Korea	−0.25***			
Japan**	−0.26***			
**(3) Emotional support**				
United States	0.40***	−0.34***	–	
China	0.31***	−0.22***		
South Korea	0.32***	−0.21***		
Japan	0.30***	−0.17***		
**(4) Informational support**				
United States	0.38***	−0.29***	0.72***	–
China	0.27***	−0.17***	0.60***	
South Korea	0.31***	−0.17***	0.66***	
Japan**	0.28***	−0.10**	0.64***	
**(5) Financial support**				
United States	0.28***	−0.22***	0.51***	0.52***
China	0.23***	−0.11***	0.39***	0.48***
South Korea	0.27***	−0.11***	0.47***	0.46***
Japan	0.22***	−0.07*	0.43***	0.40***

* *p* < 0.05, ** *p* < 0.05, *** *p* < 0.001.

### Parental Support Across Cultural Groups

We conducted a 3 (support type: emotional, informational, financial) x 4 (cultural group: United States, China, South Korea, Japan) mixed model ANOVA to examine whether the cultural differences in perception of parental support vary as a function of parental support type. There was a main effect of cultural group, *F*(3,7176) = 71.21, *p* < 0.001, $\eta_{\text{p}}^{2}$ = 0.029, such that the adolescent in the United States and China perceived greater parental support than those in South Korea and Japan overall. There was also a main effect of support type, *F*(2,14352) = 599.10, *p* < 0.001, $\eta_{\text{p}}^{2}$ = 0.077, such that adolescents perceived financial support to a greater degree than other emotional or informational support from their parents. We found a significant cultural group x support type interaction, *F*(6,14352) = 116.12, *p* < 0.0001, $\eta_{\text{p}}^{2}$ = 0.046. Planned comparisons revealed that cultural group differences were significant in all three types of parental support, *F*s > 43.20, *p*s < 0.001. Specifically, American and Chinese participants did not differ in perceived emotional support with each other, *p* = 0.414, but they perceived greater emotional support than Koreans and Japanese participants, *p*s < 0.001. Koreans and Japanese did not differ in terms of emotional support, *p* = 0.910. As for informational support, Americans perceived the greatest support, followed by Chinese, Koreans, and Japanese. All pairwise differences were significant (*p*s < 0.001). Finally, as for financial support, Chinese participants perceived the greatest support (*p*s < 0.001), while Americans perceived the least with Koreans and Japanese in the middle (*p*s < 0.001). Koreans and Japanese did not differ in financial support, *p* = 0.261.

### The Relationship Between Parental Support and Psychological Well-Being Across Cultural Groups

We examined the mean levels of psychological well-being (positive self-beliefs, distress) across cultural groups as well as the association between perceived parental support (informational, financial, and emotional) and psychological well-being (positive self-beliefs, distress) using hierarchical multiple regressions. All correlations between the variables are presented in Table 3. In step 1, contrast-coded variables for cultural groups were entered. In order to compare United States to other East Asian countries, we used the contrast coefficients 3, −1, −1, −1. To compare Chinese to other East Asian countries, South Korea and Japan, we used the contrast coefficients of 0, 2, −1, −1. Finally, to compare Koreans and Japanese, we used the contrast coefficients of 0, 0, 1, −1. In step 2, we entered informational, financial, and emotional parental support. In step 3, the two-way interaction between three parental support types and cultural groups were entered. Initial analyses included gender and age but revealed no significant effects, therefore, were excluded these variables from further analyses.

#### The Relationship Between Parental Support and Positive Self-Beliefs

In Step 1, there were significant main effects of cultural groups [*R*^2^ = 0.21, *F*(3,7174) = 631.30, *p* < 0.001]. Specifically, being American, *B* = 0.119, *SE* = 0.004, β = 0.287, *t* = 26.66, *p* < 0.001 was associated with higher levels of positive self-beliefs relative to other East Asians. In addition, Chinese reported higher levels of positive self-beliefs than other East Asians, Koreans and Japanese, *B* = 0.176, *SE* = 0.006, β = 0.339, *t* = 30.69, *p* < 0.001. Koreans were higher in positive self-beliefs than Japanese, *B* = 0.211, *SE* = 0.009, β = 0.273, *t* = 24.38, *p* < 0.001. In Step 2, emotional parental support (*B* = 0.136, *SE* = 0.011, β = 0.183, *t* = 13.25, *p* < 0.001), informational support (*B* = 0.087, *SE* = 0.012, β = 0.117, *t* = 8.46, *p* < 0.001), and financial support independently and positively predicted levels of positive self-beliefs *[B* = 0.082, *SE* = 0.010, β = 0.095, *t* = 8.288, *p* < 0.001, Δ*R*^2^ = 0.10, *F*(3,7171) = 363.75, *p* < 0.001]. In Step 3, the inclusion of two-way interaction between the three types of parental support and cultural groups did not contribute to additional increase in the variance (Δ*R*^2^ = 0.001, *p* = 0.750), indicating that emotional, informational, and financial support predicted levels of positive self-beliefs similarly across cultural groups (Table 4).

**TABLE 4:** Hierarchical multiple regression predicting positive self-beliefs.

**Predictor**	**β**	***SE***	***t***	**CI_95__%_ LL**	**CI_95__%_ UL**	**Δ*R*^2^**	**Δ*F***
***Step 1***						0.21**	631.30**
C1	0.29	0.00	26.66**	0.11	0.13		
C2	0.34	0.01	30.69**	0.16	0.19		
C3	0.27	0.01	24.38**	0.19	0.23		
***Step 2***						0.10**	363.75**
C1	0.26	0.00	24.61**	0.10	0.11		
C2	0.29	0.01	28.18**	0.14	0.16		
C3	0.26	0.01	24.66**	0.19	0.22		
Emotional PS	0.18	0.01	13.25**	0.11	0.15		
Informational PS	0.12	0.01	8.46**	0.07	0.11		
Financial PS	0.10	0.01	8.29**	0.06	0.10		
***Step 3***						0.001	0.66
C1	0.25	0.02	5.29**	0.07	0.14		
C2	0.28	0.03	4.69**	0.08	0.21		
C3	0.22	0.04	4.02**	0.09	0.25		
Emotional PS	0.18	0.01	12.45**	0.11	0.16		
Informational PS	0.12	0.01	7.38**	0.06	0.11		
Financial PS	0.09	0.01	7.00**	0.06	0.10		
C1 x Emotional PS	0.03	0.01	0.65	−0.01	0.02		
C1 x Informational PS	0.05	0.01	0.91	−0.01	0.02		
C1 x Financial PS	−0.08	0.01	–1.55	−0.02	0.00		
C2 x Emotional PS	0.04	0.01	0.79	−0.01	0.02		
C2 x Informational PS	−0.04	0.01	–0.74	−0.03	0.01		
C2 x Financial PS	0.01	0.01	0.21	−0.02	0.02		
C3 x Emotional PS	−0.01	0.01	–0.20	−0.03	0.02		
C3 x Informational PS	0.00	0.01	0.06	−0.03	0.03		
C3 x Financial PS	0.05	0.01	0.95	−0.01	0.04		

** *p* < 0.001. C1 = United States vs. China, South Korea, Japan; C2 = China vs. South Korea, Japan; C3 = South Korea vs. Japan; PS = parental support.

#### The Relationship Between Parental Support and Distress

In Step 1, In Step 1, there were significant main effects of cultural groups [*R*^2^ = 0.064, *F*(3,7040) = 160.64, *p* < 0.001]. Specifically, being American, *B* = 0.055, *SE* = 0.006, β = 0.117, *t* = 9.92, *p* < 0.001 was associated greater distress relative to other East Asians. In addition, Chinese reported higher levels of distress than other East Asians, Koreans and Japanese, *B* = 0.038, *SE* = 0.007, β = 0.066, *t* = 5.43, *p* < 0.001. Koreans reported lower levels of distress than Japanese, *B* = −0.148, *SE* = 0.011, β = −0.170, *t* = −13.79, *p* < 0.001. In Step 2, perceived emotional (*B* = -0.157, *SE* = 0.013, β = -0.188, *t* = -12.14, *p* < 0.001) and informational parental support (*B* = −0.039, *SE* = 0.013, β = −0.046, *t* = -2.90, *p* = 0.004) independently and negatively predicted levels of distress, but financial support did not, *B* = -0.01, β = -0.011, *SE* = 0.013, *t* = −0.85, *p* = 0.393 [Δ*R*^2^ = 0.05, Δ*F*(3,7037) = 132.47, *p* < 0.001]. In Step 3, the additional interaction terms significantly contributed to the change in variance [Δ*R*^2^ = 0.003, Δ*F*(9,7028) = 2.34, *p* = 0.012], but none of the two-way interactions between cultural groups and three types of parental support were significant (Table 5).

**TABLE 5:** Hierarchical multiple regression predicting distress.

**Predictor**	**β**	***SE***	***t***	**CI_95__%_ LL**	**CI_95__%_ UL**	**Δ*R*^2^**	**Δ*F***
***Step 1***						0.06***	160.64***
C1	0.12	0.01	9.92^∗∗∗^	0.04	0.07		
C2	0.07	0.01	5.43^∗∗∗^	0.02	0.05		
C3	−0.17	0.01	−13.79***	−0.17	−0.13		
***Step 2***						0.05***	132.47***
C1	0.14	0.01	11.65***	0.05	0.08		
C2	0.09	0.01	7.71***	0.04	0.07		
C3	−0.16	0.01	−13.57***	−0.16	−0.12		
Emotional PS	−0.19	0.01	−12.14***	−0.18	−0.13		
Informational PS	−0.05	0.01	–2.90^∗∗^	−0.06	−0.01		
Financial PS	−0.01	0.01	–0.85	−0.04	0.01		
***Step3***						0.003*	2.34*
C1	0.34	0.03	6.29***	0.11	0.21		
C2	0.16	0.04	2.26^∗^	0.01	0.17		
C3	−0.12	0.05	–1.95	−0.21	0.00		
Emotional PS	−0.19	0.01	−11.52***	−0.19	−0.14		
Informational PS	−0.04	0.02	−2.46^∗^	−0.07	−0.01		
Financial PS	−0.01	0.01	–0.81	−0.04	0.02		
C1 x Emotional PS	−0.08	0.01	–1.32	−0.03	0.01		
C1 x Informational PS	−0.08	0.01	–1.15	−0.03	0.01		
C1 x Financial PS	−0.06	0.01	–0.99	−0.02	0.01		
C2 x Emotional PS	0.01	0.01	0.20	−0.02	0.02		
C2 x Informational PS	−0.06	0.01	–0.96	−0.04	0.01		
C2 x Financial PS	−0.02	0.01	–0.28	−0.03	0.02		
C3 x Emotional PS	0.05	0.02	0.83	−0.02	0.05		
C3 x Informational PS	−0.09	0.02	–1.52	−0.06	0.01		
C3 x Financial PS	−0.01	0.02	–0.15	−0.04	0.03		

* *p* < 0.05, ** *p* < 0.01, *** *p* < 0.001. C1 = United States vs. China, South Korea, Japan; C2 = China vs. South Korea, Japan; C3 = South Korea vs. Japan; PS = parental support.

### The Effect of Parental Emotional Support on Distress Controlling for Self-Esteem

In order to test whether the positive association between emotional parental support and well-being is due to the self-esteem for American participants as found in Uchida et al. (2008), we regressed distress on perceived emotional parental support with cultural group as a moderator. That is, to determine whether self-esteem mediated the relationship between parental emotional support and distress differently across cultural groups, we conducted moderated mediation analysis, using the PROCESS Model 58 (Hayes, 2013). The results showed that the index of moderated mediation was significant (index of moderated mediation = −0.05, *SE* = 0.01, 95% CI[−0.08, −0.02]. Specifically, indirect effect of self-esteem on the relationship between emotional support and distress was significantly stronger for Americans (β = −0.11, *SE* = 0.01, 95% CI[−0.14, −0.08]) than for East Asians (β = −0.06, *SE* = 0.01, 95% CI[−0.07, −0.05]). Thus, self-esteem partially mediated the effect of parental emotional support on distress for not only American adolescents but also for East Asian adolescents. This may indicate that self-esteem is a critical element that contributes to adaptive functioning of adolescents regardless of their cultural background, and particularly for American adolescents.

## Discussion

The current study examined cultural similarities and differences in perceived parental support among adolescents from the United States, China, South Korea, and Japan. As hypothesized, American adolescents reported receiving higher levels of emotional support from their parents than Korean and Japanese adolescents, with Chinese adolescents reporting levels of support that were not different from those of the Americans. Unexpectedly, data for perceived informational support followed the same pattern, with the United States and Chinese adolescents reporting that they received more information and advice from their parents than Korean and Japanese adolescents. In contrast to the pattern observed for emotional and informational support, East Asian adolescents, and particularly Chinese teens, tended to report higher levels of perceived financial support from their parents than the United States teens. Taken together, these data indicate that across cultural contexts, adolescents report being on the receiving end of substantial levels of perceived parental support. The type of perceived support, however, differs by cultural contexts, with predominantly emotional and informational support provision in the United States, financial support in Japan and South Korea, and a combination of emotional and tangible support in China. These results also suggest that the tendency of the social support researchers to group informational and financial support under the rubric of tangible support may conceal important differences between the two. The correlation between informational and financial parental support was 0.52 for Americans, 0.46 for Koreans, 0.40 for Japanese, and 0.48 for Chinese (all *p* < 0.001), suggesting that although these two types of support are positively associated with each other, they are far from identical.

Also as predicted, Chinese adolescents reported higher levels of parental support than teens from other East Asian contexts. This observation builds on prior literature suggesting that contemporary Chinese culture fosters higher levels of parental warmth than other East Asian cultures (Farruggia et al., 2004). These within East-Asia differences deserve more attention from researchers as they may help us understand cultural changes in China versus South Korea and Japan. Given the literature on downstream consequences of parental support (Orth et al., 2012), these differences may contribute to future patterns of cultural divergence between these contexts. Of course, our data do not allow us to conclude that this pattern is due to the One Child policy but future studies may examine cultural factors (e.g., family size) that contribute to these differences over time.

Another finding emerging from the current study is that perceived parental support influenced self-beliefs and distress levels similarly across cultural settings. When predictors of positive self-beliefs were examined, we found that adolescents from the United States, South Korea, China, and Japan were equally likely to benefit from receiving affection, information and money from their parents. These results are in line with prior work on cultural similarities in the impact of social support on psychological functioning. Overall, these data stand in contrast to prior work conducted with adults, which indicates that Chinese cultural context is more likely to foster preference for advice than European American cultural contexts (e.g., Xu and Burleson, 2004). They may indicate that American cultural norms discourage informational support in egalitarian interactions between adults (e.g., married couples, friends, colleagues), but may actually encourage its use in inherently hierarchical parent-child interactions. Given the crucial role of parental support among adolescents, these results illustrate the need for doing more research on culture and social support with this population rather than assuming that the patterns observed for adults will hold for adolescents.

Another interesting finding was that the beneficial impact of financial support was limited to fostering self-beliefs and did not extend to ameliorating distress. Notably, the reliability of our measure of financial support tended to be lower than those for emotional and informational support, likely limiting the associations of this measure with other variables.^1^ Hence, it is possible that this pattern provides us with a conservative estimate of the association of financial support with distress. Alternatively, this pattern may be due to the fact that, unlike emotional and informational forms of support, financial support does not require face-to-face interactions between parents and teens. Knowing that one’s parents invest money in tutoring or allowance may effectively signal to the adolescents that their parents see them as important and valuable. This understanding may be beneficial to their self-esteem, self-efficacy, and optimism. However, it appears that it does little to prevent adolescents from feeling anxious or depressed. These results suggest that although all types of perceived parental support examined in this study appear functional, some types of support are particularly well-suited for particular goals (i.e., emotional and informational support promoting emotional well-being). Furthermore, considering that some researchers suggest excess in parental support may harm adolescents’ well-being (Coccia et al., 2012), more research is needed to examine the potentially negative impact of different types of parental support on well-being.

### Limitations and Future Directions

This study was not without its limitations. First of all, the study relied on global self-reports of perceived support from adolescents. We know that perceived support does not always map onto actual support. Comparing perceptions of support-providers with those reported by people on the receiving end of their support has proven to be fruitful in studies of support among adults (e.g., Bolger et al., 2000). Additionally, it is well-known that global or retrospective reports do not always map onto in-the-moment experiences (e.g., Schwarz et al., 2009). Future studies should examine parents’ reports of their support to determine whether adolescents’ and parents’ reports paint the same picture. It would also be valuable to attempt to capture parental support of adolescents as it occurs in their daily lives by using observational studies or daily diaries of support. Finally, emerging literature suggests that culture can affect the relationship between supportive relationships and proinflammatory activity (Chiang et al., 2013). In the future, it would be important to examine physiological manifestations of perceived parental support among adolescents.

Furthermore, the types of parental support examined in this study were limited to emotional, informational and financial support. Although this examination of three distinct types of support represents a significant advance over prior literature on parental support, the current study nonetheless overlooks other types of support that may be important. In particular, the study did not examine certain types of support that may be particularly important in East Asian cultural contexts, such as taking care of chores, cooking special meals, seeking network connections to help the teen, or simply being present during stressful times. In many East Asian contexts, it is common to see parents accompanying their adolescent children to important exams and waiting for them to show dedication and support (Davey et al., 2007). This is but one example of implicit parental support in action. Although it can be challenging to capture implicit support due to its “invisible” quality, future studies should attempt to study it in this population.

Another important limitation is that this study did not directly measure cultural factors that may contribute to parental supports. Instead, we relied on prior studies’ characterizations of these cultural contexts. Future work should directly assess factors such as independence and interdependence and concerns about autonomy and burden in order to better understand factors that contribute to cultural differences in parental support.

Finally, although every effort was made to obtain representative samples of high school students in four different cultural contexts, it is unclear how these data generalize to younger or older teens and those from other cultural contexts. Future work on this topic should aim to examine developmental changes in perceived parental support and its utility for adolescents’ well-being. It is likely that as children age, support remains important, yet, particular types of support that are beneficial for growing children may depend on their age. Examining a broader range of age groups and cultural groups remains an important task for studies of culture and parental support.

## Conclusion

In summary, this study is among the first to examine cross-cultural similarities and differences in perceived parental support among adolescents. It adds to the literature by adding specificity. Instead of lumping different types of parental support together, this study examined levels of three different types of support and their impact of positive self-beliefs and psychological distress. This was an important advance, as we found that the observed patterns for emotional, informational and financial support differed from each other. Similarly, instead of examining broad differences between North American and East Asian cultural contexts that are so popular in contemporary research in cultural psychology, this study focused on the differences between the three East Asian cultural contexts with distinct histories of cultural shaping of families and parenting. This effort resulted in data suggesting that perceived parental support in China systematically differs from that in South Korea and Japan. Hence, future work on this topic can no longer assume that findings based in China would hold for Japanese or Korean cultural contexts and vice versa. Instead, it invites us to examine cultural shaping of parenting in each of the individual cultural contexts, systematically assessing cultural values, beliefs, institutions and practices that foster some types of support over others.

Taken together, these findings suggest that perceived parental support is very important for psychological well-being of adolescents across cultural contexts. Yet, we also observed that culture shapes not only the levels of different types of support, but also their impact on some aspects of psychological well-being of adolescents. These results have important implications not only for researchers interested in culture and social support, but also for the larger community. Researchers need to remember that both age and cultural contexts can shape the nature and impact of social support. Practitioners such as counselors or parent educators can benefit from exploring cultural norms that impact the lives of families that come to them for help. Finally, parents can benefit from learning that their efforts to support their teens by encouraging them, giving them advice and investing in them financially have important dividends for their teens’ psychological well-being.

## Data Availability Statement

The datasets generated for this study are available on request to the corresponding author.

## Ethics Statement

The studies involving human participants were reviewed and approved by Byungsoo Kim, Korea University. Written informed consent to participate in this study was provided by the participants’ legal guardian/next of kin.

## Author Contributions

YC structured the framework of the manuscript and led the writing of the manuscript. EC analyzed the data and wrote the results section of the manuscript. I-JC collected the data and participated in data analysis.

## Conflict of Interest

The authors declare that the research was conducted in the absence of any commercial or financial relationships that could be construed as a potential conflict of interest.

## Footnotes

1 The main regression analyses conducted with each item of parental financial support separately yielded similar results.
