# Corrigendum: Perceived Parental Support and Adolescents' Positive Self-Beliefs and Levels of Distress Across Four Countries

**DOI:** 10.3389/fpsyg.2020.00532

**Published:** 2020-03-31

**Authors:** Yulia E. Chentsova Dutton, In-Jae Choi, Eunsoo Choi

**Affiliations:** ^1^Department of Psychology, Georgetown University, Washington, DC, United States; ^2^National Youth Policy Institute, Seoul, South Korea; ^3^Department of Psychology, Korea University, Seoul, South Korea

**Keywords:** adolescents, parental support, culture, positive self-beliefs, distress

In the published article, there was an error in the affiliation of the second and third author. The affiliations of In-Jae Choi and Eunsoo Choi are switched. In-Jae Choi's affiliation is **“National Youth Policy Institute, Seoul, South Korea”** and Eunsoo Choi's affiliation is **“Department of Psychology, Korea University, Seoul, South Korea.”**

The authors apologize for this error and state that this does not change the scientific conclusions of the article in any way. The original article has been updated.

