# Supplementary material for: Perceived Parental Support and Adolescents’ Positive Self-Beliefs and Levels of Distress Across Four Countries
Source: Front Psychol. 2020 Mar 3;11:353. doi: 10.3389/fpsyg.2020.00353 (PMC7079630; doi:10.3389/fpsyg.2020.00353)
Supplement: Supplementary file 1 [file Table_1.DOCX]

# **Appendix A**

**Items measuring positive self-beliefs, distress, perception of parental support (emotional, informational, financial)**

**Positive self-beliefs**

What do you think about yourself. (1 = disagree, 2 = somewhat disagree, 3 = somewhat agree, 4 = agree)

1. Self-esteem
2. I am a valuable person.
3. I have a good personality.
4. I can cooperate well with others most of the time.
5. I evaluate myself positively.
6. I am satisfied with myself.
7. Self-efficacy
8. I can do pretty much anything if I make an effort.
9. There are many things I can do.
10. I can overcome hardships.
11. Optimism
12. I believe my dreams will come true.
13. I can get over easily when I fail to reach goals.
14. I feel I’m highly capable.

**Distress**

Did you ever experience the following feelings during the past week? (1 = Never, 2 = Sometimes, 3 = Occasionally, 4 = Often)

1. Depression
2. Lonely
3. Gloomy
4. Empty
5. Anxiety
6. Edgy and agitated
7. Worried for no reason
8. Nervous
9. Anxious
10. Confused and annoyed
11. Anger
12. Wanted to punch and hurt someone
13. Wanted to break something
14. Wanted to yell and throw something

**Perception of parental support**

What do you think about your parents/ guardians? (1 = disagree, 2 = somewhat disagree, 3 = somewhat agree, 4 = agree)

1. Emotional support
2. They understand me well.
3. They listen to my problems.
4. They help me when I am having difficult times.
5. Informational support
6. They give me advice on my career options.
7. They give me useful advice on my studies.
8. They teach me the right attitudes about studying and life.
9. Financial support
10. They give me allowance.
11. They support me so that I can live without financial worries.
12. They buy me necessary items for study.

# **Appendix B**

Descriptive statistics for sub-factors of positive self-beliefs (self-esteem, self-efficacy, optimism) (Table B1) and distress (depression, anxiety, anger) (Table B2)

Table B1. Means and standard deviations of positive self-beliefs

|  | US | China | Korea | Japan | *F*(3, 7189) |
| --- | --- | --- | --- | --- | --- |
| Self-esteem | 3.37_a_  (0.59) | 3.18_d_  (0.55) | 2.86_b_  (0.60) | 2.32_c_  (0.62) | 649.20*** |
| Self-efficacy | 3.51_a_  (0.59) | 3.20_d_  (0.62) | 2.87_b_  (0.57) | 2.46_c_  (0.68) | 619.82*** |
| Optimism | 2.85_a_  (0.75) | 2.97_d_  (0.65) | 2.68_b_  (0.57) | 2.35_c_  (0.65) | 208.36*** |
| Total | 3.24  (0.55)_a_ | 3.12  (0.51)_b_ | 2.80  (0.48)_c_ | 2.38  (0.55)_d_ | 632.07^***^ |

Note. *** *p* < .001. Standard deviations appear in parentheses bellow means. Means with differing subscripts within rows are significantly different at the p < .05 based on Sheffé’s post hoc paired comparisons.

Table B2. Means and standard deviations of distress

|  | US | China | Korea | Japan | *F*(3, 7189) |
| --- | --- | --- | --- | --- | --- |
| Depression | 1.94_a_  (0.90) | 2.14_b_  (0.81) | 1.93_a_  (0.72) | 2.22_b_  (0.96) | 49.95^***^ |
| Anxiety | 2.23_b_  (0.81) | 1.93_c_  (0.70) | 1.66_a_  (0.66) | 1.91_c_  (0.83) | 188.43^***^ |
| Anger | 2.05_b_  (0.89) | 1.72_c_  (0.74) | 1.42_a_  (0.61) | 1.79_c_  (0.82) | 247.00^***^ |
| Total | 2.07_b_  (0.71) | 1.93_c_  (0.63) | 1.67_a_  (0.56) | 1.97_c_  (0.75) | 161.49^***^ |

Note. *** *p* < .001. Standard deviations appear in parentheses bellow means. Means with differing subscripts within rows.
